# SMALF: miRNA-disease associations prediction based on stacked autoencoder and XGBoost

**DOI:** 10.1186/s12859-021-04135-2

**Published:** 2021-04-28

**Authors:** Dayun Liu, Yibiao Huang, Wenjuan Nie, Jiaxuan Zhang, Lei Deng

**Affiliations:** 1 School of Computer Science and Engineering, Central South University, Hunan, 410083 China; 2 Department of Cognitive Science, University of California San Diego, La Jolla, 92093 USA

**Keywords:** miRNA-disease associations, Stacked autoencoder, Latent feature, XGBoost

## Abstract

**Background:**

Identifying miRNA and disease associations helps us understand disease mechanisms of action from the molecular level. However, it is usually blind, time-consuming, and small-scale based on biological experiments. Hence, developing computational methods to predict unknown miRNA and disease associations is becoming increasingly important.

**Results:**

In this work, we develop a computational framework called SMALF to predict unknown miRNA-disease associations. SMALF first utilizes a stacked autoencoder to learn miRNA latent feature and disease latent feature from the original miRNA-disease association matrix. Then, SMALF obtains the feature vector of representing miRNA-disease by integrating miRNA functional similarity, miRNA latent feature, disease semantic similarity, and disease latent feature. Finally, XGBoost is utilized to predict unknown miRNA-disease associations. We implement cross-validation experiments. Compared with other state-of-the-art methods, SAMLF achieved the best AUC value. We also construct three case studies, including hepatocellular carcinoma, colon cancer, and breast cancer. The results show that 10, 10, and 9 out of the top ten predicted miRNAs are verified in MNDR v3.0 or miRCancer, respectively.

**Conclusion:**

The comprehensive experimental results demonstrate that SMALF is effective in identifying unknown miRNA-disease associations.

## Background

Human cells contain a variety of non-coding RNAs. MicroRNAs(miRNAs) are a set of short non-coding RNA, with about 20–25 nucleotides in length, which play an essential role in various biological processes of living organisms [1]. In 1993, the first miRNA lin-4 was discovered in elegans [2]. However, this discovery didn’t catch researchers’ attention at that time, and people used to see miRNAs as the “Dark Matter”. Now, a substantial number of miRNAs have been found in animals, plants, viruses, and humans. Mounting evidences have shown that miRNAs participate in cell proliferation, cell division, cell death, cell differentiation, hematopoiesis, and neural development [3].

Besides, miRNAs have been identified to regulate gene expression post-transcriptionally by affecting the translation of mRNA[4], which means the dysregulation of miRNAs may be associated with kinds of diseases by affecting gene expression. Studies have validated that miRNAs are closely related to diseases [5, 6]. For example, chronic lymphocytic leukemia(CLL) results from miR-15 and miR-16 by controlling the antiapoptotic B-cell lymphoma protein BCL-2 in B cells [7]. Iorio proposed the abnormal expression of miR-21, miR-125b, miR-145, and miR-155 are involved in human breast cancer [8]. Kozaki observed oral squamous cell carcinomas(OSCC) are associated with the following miRNAs. miR-34b, miR-137,miR-193a, and miR-203, which were silenced by aberrant DNA methylation [9]. Glioblastoma multiform(GBM) pathogenesis are shown to be associated with the deregulation of miR-21 [10]. Also, the decreased expression of APP and BACE1 regulated by miR-9, miR-29a, and miR-29b-1 may increase the occurrence of Alzheimer’s ailment [11]. Based on the research above, predicting miRNA-disease association is apparently a valuable field to research. It provides a better understanding of the pathogenesis of diseases, and contributes a lot to prevent and diagnose illnesses.

In earlier studies, researchers devoted to identifying miRNA-disease association using conventional biological experiments, which are pricey, time-consuming, laborious, and easy to fail. In those studies, a mass of biological datasets still has been collected. Therefore, establishing an effective computational model with high accuracy to predict the connection with miRNAs and diseases is essential. Nowadays, machine learning, deep learning, and methods that combine the above algorithms are widely applied in proposed computational models, mainly relying on the assumption that miRNAs with similar functions are nearly related to similar diseases [12]. For example, Chen et al.[13] built a random walk-based computational model named RWRMDA to reveal miRNA-disease association. Xuan et al. [14] presented a network-based model named MIDP, which considered the prior information and the structure of different categories of network nodes, diminished the negative effect of noisy data effectively and performed better than Chen’s RWRMDA [13]. Chen et al. improved their original work to create a new model, GRMDA [15], using graph regression synchronously on miRNA, disease, and association graph, while combining with Partial Least-Squares to reduce the noise. Jiang et al. [16] proposed ICFMDA to uncover the unknown relationship between miRNA and diseases through using the similarity matrices to adjust the weight of the bipartite network of miRNA and diseases, implementing a collaborative filtering algorithm to suggest miRNA or diseases to each other. You et al. [17] put forward PBMDA using the similarity of miRNA and diseases as subgraphs to construct a heterogeneous graph, applying a depth-first search algorithm to traverse the graph’s paths to find the possible connection between miRNA and disease.

The above approaches are generally based on graphs to predict the relationship between miRNA and diseases. This way can effectively dig out the potential, deep-seated, unknown relationship between miRNA and disease from the existing relationship between miRNA and disease, and the use of graphs can more clearly understand the connection between miRNA and disease. However, methods based on graphs are easily biased towards miRNAs or diseases which have many known associations. For diseases with few known associations, it is difficult for them to fully obtain accurate miRNAs candidates because sparse links limit information propagation. Meanwhile, with the spring up of machine learning and deep learning, more and more machine learning and deep learning algorithms are utilized for miRNA-disease prediction. Yao et al. [18] used random forest for feature selection and selected the top 100 features to use random forest regression to score the connection between miRNA and disease. Zheng et al. [19] raised a machine learning-based model named MLMDA, which adopted a deep auto-encoder neural network to extract features and the random forest classifier to deduce miRNA-disease interaction. Zhao et al. [20] utilized k-means clustering in data-processing to balance the positive and negative sample and presented ABMDA implemented by boosting algorithm that iterates the weak classifier, decision tree, to improve the accuracy of classification to know the potential miRNA-disease interaction. Wang et al. [21] first integrated the miRNA sequence information with miRNA and disease similarity to extract features, and they applied the logistic tree model to classify the relationship between miRNA and disease, with 90.54% AUC value. Zhou et al. [22] constructed a novel model GBDT-LR using GDBT to extract latent features efficiently and logistic regression to score the disease-miRNA interaction. Zhang et al.[23] obtained two splicing matrices from the similarity matrix and association matrix of disease and miRNA, and then adopted two variational autoencoders to predict the unknown miRNA-disease interaction. Xuan et al. [24] proposed CNNMDA constructed by CNN to train the local and global features acquired from the two embedding layers learn from the association between miRNA and disease respectively to expose the relationship between miRNA and disease. Chen et al. [25] presented a model that can easily extend to higher dimension datasets called LRSSLMDA implemented by Laplacian regulation and L1-norm to optimize the function to get the possible connection between disease and miRNA. Fu et al. [26] implemented DeepMDA which uses stacked autoencoder to extract features and applies a 3-layer neural network to identify the connection between miRNA and disease. Li et al.[27] presented MCMDA using the SVT algorithm to complete the matrix to obtain an updated miRNA-disease association matrix to predict miRNA and disease connection. Zhao et al. [28] put forward the Spy and Super Cluster strategy to uncover the interaction between disease and miRNA based on the established miRNA-disease association. Furthermore, Luo et al. [29] put forward KPLMS to reveal the potential connection of miRNA and disease by combining miRNA and disease through Kronecker product into the whole space and using regularized least squares to predict miRNA-disease interaction. Also, a novel model presented by Gong et al. [30] utilizing random forest to train the features obtained from miRNA-disease association matrix and disease description graph is designed for miRNA-disease association prediction.

We can regard miRNA-disease association prediction as a miRNA-disease recommendation system. There are complex potential factors hidden under the miRNA-disease association matrix. Unearthing these potential factors can help accurately predict miRNA-disease associations. Hence, we present a novel approach to extract latent features from the original miRNA-disease association matrix. In this work, we develop a calculation framework called SMALF that utilizes stacked autoencoder and XGBoost to infer unknown miRNA-disease associations by integrating latent features and similarities. Stacked autoencoder is an unsupervised learning model that can extract latent features from the input information [31]. XGBoost is a representative of the boosting algorithm, which can effectively enhance the classification effect by integrating many weak classifiers to generate a robust classifier[32]. In SMALF, firstly, we use stacked autoencoders to extract miRNA latent feature and disease latent feature from the original miRNA-disease association matrix. Next, cascade latent features and similarities to obtain feature vectors. Finally, adopt the XGBoost model to complete the classification prediction. To evaluate the performance of SMALF, we perform cross-validation experiments. The AUC of SMALF reached 0.9503, which is much higher than other models. Simultaneously, the top 10 miRNAs predicted for hepatocellular carcinoma, colon cancer, and breast cancer were 10, 10, and 9 verified in other databases, respectively. All in all, SMALF can effectively predict miRNA-disease associations.

## Results and discussion

### The performance of SMALF based on five-fold cross-validation

In this section, to validate the ability of SMALF to infer unknown miRNA-disease associations, we adopt the five-fold cross-validation in our experiment. The dataset is randomly divided into five subsets, then four subsets are selected for training and one subset for testing. This process is repeated until all subsets have been used for the test set. In classification problems, the ROC curve is an important method to evaluate model performance. The horizontal coordinate of the ROC curve is the false positives rate (FPR), and the vertical coordinate being the true positives rate (TPR).FPR and TPR is given by the following formulas:

1 $$\begin{aligned} FPR= & {} \frac{{\mathrm{{FP}}}}{{TN + FP}} \end{aligned}$$

2 $$\begin{aligned} TPR= & {} \frac{{\mathrm{{TP}}}}{{TP + FN}} \end{aligned}$$

**Fig. 1:**
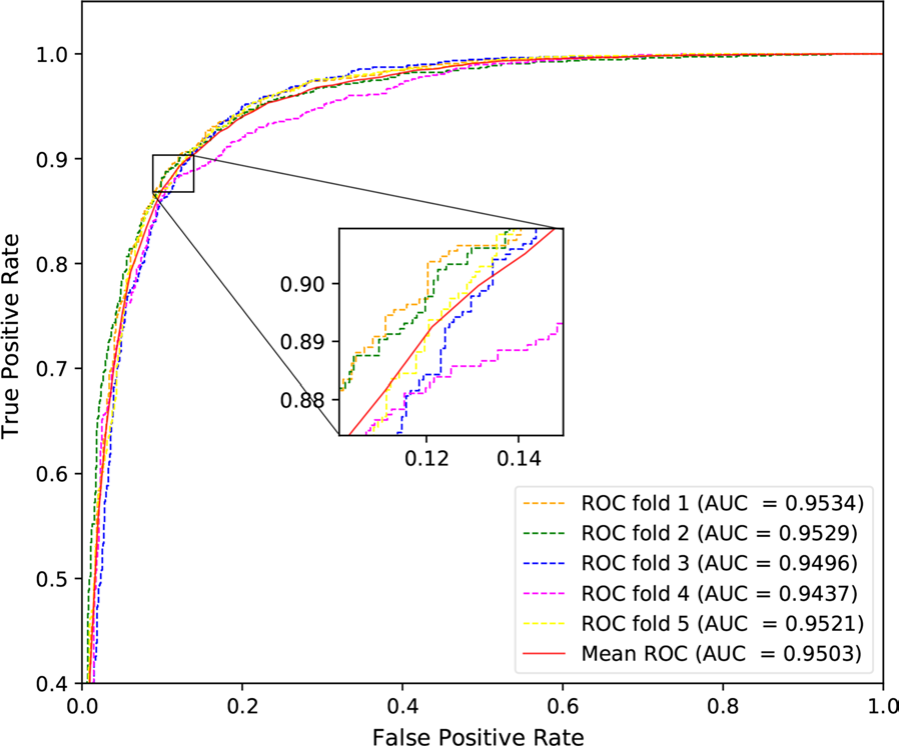
The ROC curve of SMALF based on five-fold cross validation

where TP and TN are the numbers of miRNA-disease association pairs and non-association pairs which are correctly identified, respectively; FP and FN are the numbers of miRNA-disease association pairs and non-association pairs which are incorrectly identified, respectively. This paper selects the AUC value as the main evaluation index. The AUC value is the area under the ROC curve, and its value is between 0 and 1. We can regard AUC as the probability that a positive sample is ranked higher than a negative sample in a test. Generally, if a model has good performance, its AUC is usually high as well.

Figure 1 shows the performance of SMALF based on five-fold cross-validation. As we can see from Fig. 1, AUCs of SMALF are 0.9534,0.9529,0,9496,0.9437,0.9521, respectively. The average AUC value is 0.9503. The results indicate that SMALF has good performance in inferring unknown miRNA-disease associations.

### Analysis the dimensionality of latent feature

In SMALF, we use stacked autoencoders to obtain latent feature from the original miRNA-disease association matrix. If the dimensionality of the latent feature is too short, the model cannot fully learn the association between miRNA and disease. If the dimensionality of the latent feature is too long, the risk of overfitting will increase. In this section, in order to study the impact of the dimensionality of the latent feature on the model, we set the dimensionality of latent feature to 8, 16, 32, 64, 128 for experimental comparison.

**Table 1:** The AUC, AUPR, Precision, Recall,F1_score and Accuracy of latent feature in different dimensions

Dimensionality	AUC	AUPR	Precision	Recall	F1_score	Accuracy
8	0.9371	0.9371	0.8623	0.8756	0.8689	0.8678
16	0.9436	0.9392	0.8682	0.8841	0.8760	0.8748
32	0.9452	0.9404	0.8748	0.8828	0.8788	0.8781
**64**	**0.9503**	0.9472	**0.8808**	**0.8931**	**0.8868**	**0.8860**
128	0.9495	**0.9479**	0.8795	0.8869	0.8831	0.8825

Bold values represent relatively good performance

The experimental results are shown in Table 1. From Table 1, we can see that the model achieves the optimal AUC value when the dimensionality of latent feature is 64. Therefore, in this study, we set the dimensionality of latent feature to 64.

### Analysis effects of feature vectors

How to construct feature vectors to represent per miRNA-disease has an essential role in inferring unknown miRNA-disease associations. In SMALF, we combine similarity data and latent features to represent per miRNA-disease. To verify whether our combined strategy helps infer unknown miRNA-disease associations, we designed three sets of experiments. The first set of experiments only used similarity data, directly integrating miRNA functional similarity and disease semantic similarity. We only used latent features in the second set of experiments, directly integrating the latent feature of miRNA and disease. The third set of experiments used similarity data and latent features, which was the same as SMALF.

**Fig. 2:**
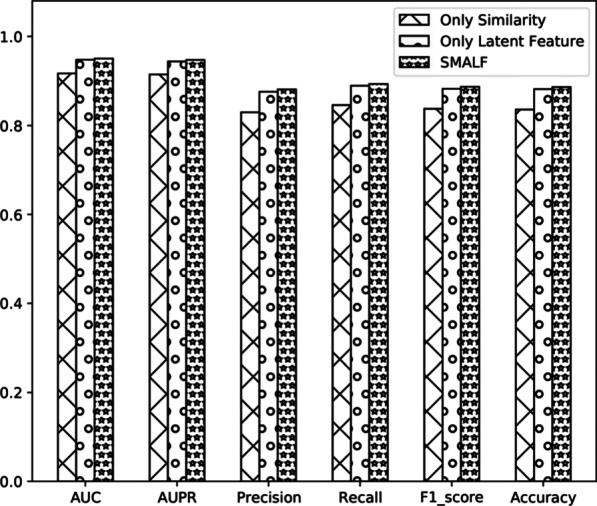
Histograms of the results of using different features vectors

**Table 2:** The AUC, AUPR, Precision, Recall,F1_score and Accuracy of using different features vectors

Feature vector	AUC	AUPR	Precision	Recall	F1_score	Accuracy
Only Similarity	0.9167	0.9145	0.8297	0.8458	0.8376	0.8359
Only Latent Feature	0.9476	0.9437	0.8756	0.8891	0.8822	0.8815
**SMALF**	**0.9503**	**0.9472**	**0.8808**	**0.8931**	**0.8868**	**0.8860**

Bold values represent relatively good performance

The results are shown in Table 2 and Fig. 2, AUCs of models using similarity data, only using latent feature, and combining similarity data and latent feature are 0.9161, 0.9467, and 0.9503. In summary, combining similarity data and latent feature gets better performance than only using similarity data or latent feature in inferring potential miRNA-disease associations.

### Comparison with different classifiers

SMALF performs well on HMDD2.0 by using the XGBoost classifier. This section selected several typical classifiers (Adaboost, Random Forest, SVM) for experimental comparison. Adaboost obtains a robust classifier by integrating multiple weak classifiers, achieving good performance in many fields. Random forest integrates various decision trees, and its final output value is determined by voting on these decision trees. SVM is a classic two-class classification model, which realizes classification by maximizing the interval between two heterogeneous classes. SVM has taken excellent results on many classification problems. In the Adaboost algorithm, we choose the decision classification tree as the weak classifier, where the maximum depth of the tree is 10 and minimize samples split is 5. The remaining parameter values are the default. In the RF algorithm, we set the maximum depth of the tree to 10 and max features is 100. The remaining parameter values are default. In the SVM algorithm, we utilize RBF kernel and set C to 50. In the XGBoost algorithm, we set the number of trees to 1000, and the learning rate is 0.1. The remaining parameter values are default.

**Fig. 3:**
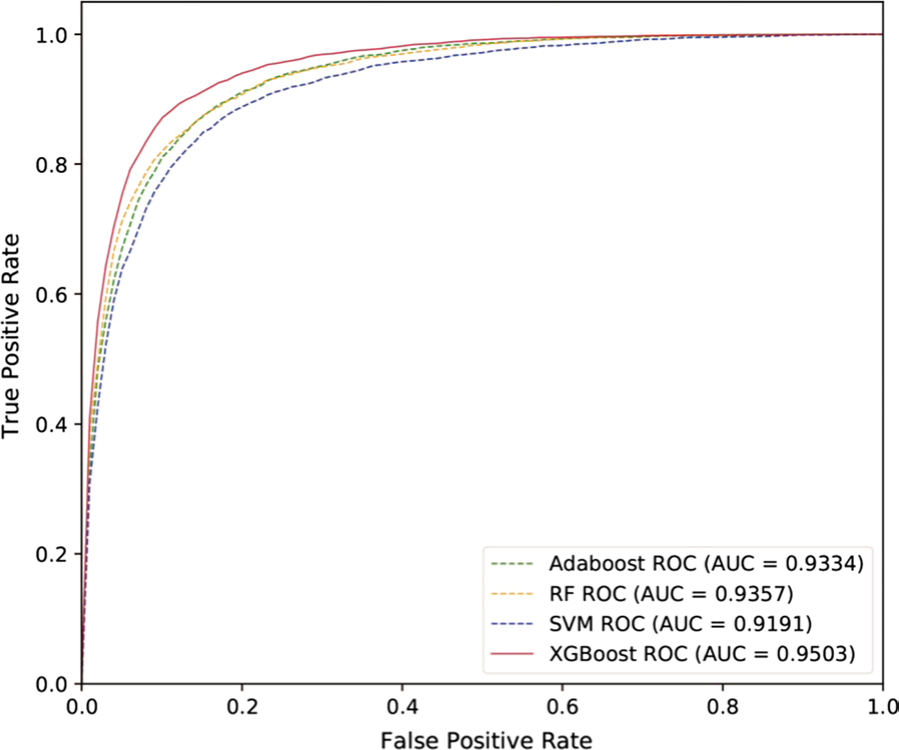
The ROC curve of SMALF with different classifiers

**Table 3:** The AUC, AUPR, Precision, Recall,F1_score and Accuracy of four classifiers

Classifier	AUC	AUPR	Precision	Recall	F1_score	Accuracy
Adaboost	0.9334	0.9301	0.8639	0.8545	0.8590	0.8597
Random Forest	0.9357	0.9334	0.8641	0.8595	0.8617	0.8620
SVM	0.9191	0.9165	0.8445	0.8499	0.8471	0.8465
**XGBoost**	**0.9503**	**0.9472**	**0.8808**	**0.8931**	**0.8868**	**0.8860**

Bold values represent relatively good performance

Table 3 and Fig. 3 show the performance of these classifiers. From Fig. 3, we can see that AUCs of Adaboost, Random Forest, SVM, XGBoost classifiers are 0.9334,0.9191,0.9357 and 0.9503, respectively. The experimental results show that XGBoost achieves much higher AUC values than the other three classifiers. Calculating miRNA functional similarity and disease semantic similarity, there are missing values in the similarity data due to the lack of biological data. Compared with other classifiers, the XGBoost algorithm handles missing values more simply and effectively.In general, the XGBoost classifier is more suitable than other classifiers for SMALF.

### Comparisons with the state-of-the-art methods

To further assess the predictive ability of SMALF, we compare the SMALF with seven other computational methods (GBDT-LR [22], LMTRDA [21], ABMDA [20], RFMDA [33], ICFMDA [16], GRMDA [15], MCMDA [27]). GDBT-LR first integrates disease similarity and miRNA similarity to represent miRNA-disease. Then, it applies GDBT to extract new features. Finally, the LR model is employed to predict miRNA-disease association. LMTRDA integrates miRNA sequence similarity, miRNA functional similarity, and disease semantic similarity. The authors creatively engage skip-gram algorithms in calculating miRNA sequence similarity. Finally, LMTRDA utilizes logistic model trees to achieve the prediction of miRNA-diseases association. ABMDA utilizes boosting algorithm which integrates many decision trees to mine miRNA-disease associations. To calculate the similarity about miRNA and disease accurately, RFMDA fuses various information and uses the random forest to realize the prediction of miRNA-disease associations.ICFMDA implements a collaborative filtering algorithm to suggest miRNA or diseases to each other.GRMDA uses graph regression synchronously on miRNA, disease, and association graph to infer miRNA-disease association. MCMDA predicts miRNA and disease association by using the SVT algorithm to obtain an updated miRNA-disease association matrix.

**Fig. 4:**
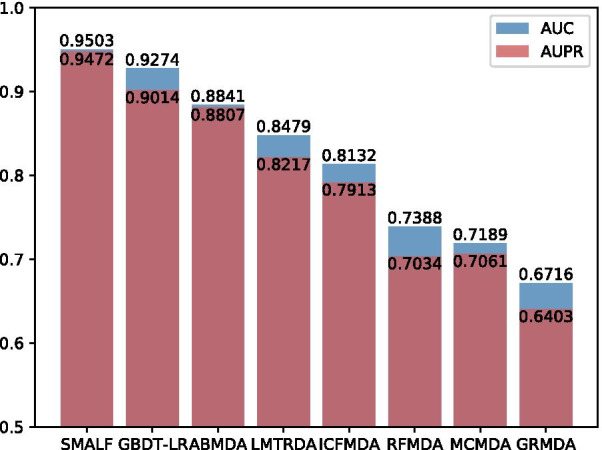
Histograms of AUC and AUPR with different computational methods

**Table 4:** The AUC, AUPR, Precision, Recall, F1_score and Accuracy of eight computational methods

Methond	AUC	AUPR	Precision	Recall	F1_score	Accuracy
**SMALF**	**0.9503**	**0.9472**	**0.8808**	0.8931	**0.8868**	**0.8860**
GBDT-LR	0.9274	0.9014	0.8315	0.8273	0.8302	0.8304
ABMDA	0.8841	0.8807	0.8152	0.7827	0.7908	0.8027
LMTRDA	0.8479	0.8217	0.8013	0.6190	0.7067	0.7327
ICFMDA	0.8132	0.7913	0.7756	0.7534	0.7643	0.7677
RFMDA	0.7388	0.7034	0.6253	**0.9548**	0.7453	0.6912
MCMDA	0.7189	0.7061	0.6801	0.6743	0.6771	0.6788
GRMDA	0.6716	0.6403	0.6284	0.6573	0.6425	0.6341

Bold values represent relatively good performance

Table 4 and Fig. 4 show experimental results for SMALF and the other seven computational methods. SMALF achieves the highest AUC value, which is 2.29% higher than the second-best model (GBDT-LR). The reason why SMALF can achieve such good results is due to using not only similarity data but also latent feature.

## Discussion

To investigate the performance of SMALF to infer unknown miRNA-disease interactions in practical application, we selected three common diseases (hepatocellular carcinoma, colon cancer, and breast cancer for case studies. In a specific disease study, we eliminated all miRNAs associated with this disease. Then we utilized SMALF to predict the remaining miRNAs’ score, getting the top 10 candidate miRNAs of this disease. Finally, we verify them by searching them in MNDR v3.0 [34] and miRCancer [35].

**Table 5:** The top 10 predicted miRNAs which may be associated with hepatocellular carcinoma

miRNA	Evidence
hsa-mir-429	MNDR v3.0 miRCancer
hsa-mir-133a	miRCancer
hsa-mir-708	MNDR v3.0 miRCancer
hsa-mir-9	miRCancer
hsa-mir-34b	MNDR v3.0 miRCancer
hsa-mir-143	MNDR v3.0 miRCancer
hsa-mir-196b	MNDR v3.0
hsa-mir-342	miRCancer
hsa-mir-184	MNDR v3.0 miRCancer
hsa-mir-539	MNDR v3.0 miRCancer

**Table 6:** The top 10 predicted miRNAs which may be associated with colon cancer

miRNA	Evidence
hsa-mir-125b	MNDR v3.0
hsa-let-7a	MNDR v3.0 miRCancer
hsa-mir-20a	MNDR v3.0
hsa-mir-29a	MNDR v3.0 miRCancer
hsa-mir-155	MNDR v3.0 miRCancer
hsa-mir-21	MNDR v3.0 miRCancer
hsa-mir-146a	MNDR v3.0 miRCancer
hsa-mir-106b	MNDR v3.0
hsa-mir-205	MNDR v3.0 miRCancer
hsa-mir-142	MNDR v3.0 miRCancer

**Table 7:** The top 10 predicted miRNAs which may be associated with breast cancer

miRNA	Evidence
hsa-mir-142	MNDR v3.0 miRCancer
hsa-mir-376a	miRCancer
hsa-mir-372	MNDR v3.0 miRCancer
hsa-mir-130b	MNDR v3.0
hsa-mir-150	MNDR v3.0 miRCancer
hsa-mir-370	MNDR v3.0 miRCancer
hsa-mir-378a	MNDR v3.0
hsa-mir-106a	MNDR v3.0 miRCancer
hsa-mir-487b	uncomfirmed
hsa-mir-186	MNDR v3.0 miRCancer

The first disease we studied is hepatocellular carcinoma. Hepatocellular carcinoma is a type of primary liver cancer that has a high mortality rate. [36] Hepatocellular carcinoma remains one of the most common and aggressive human malignancies worldwide [37, 38]. For hepatocellular carcinoma, we remove 214 miRNAs (hsa-let-7a, hsa-mir-101, hsa-mir-103a, et al.) associated with it. The remaining 281 candidate miRNAs are sent to SMALF for prediction.The results are shown in Table 5. From our study results, all the top ten miRNA candidates about hepatocellular carcinoma are confirmed in MNDR v3.0 or miRCancer.

The second disease we studied was colon cancer. Colon cancer has a high incidence in people aged 40 to 50 [39]. Colon cancer has no symptoms in its early stages, so it is straightforward to miss the diagnosis. For colon cancer, we remove 4 miRNAs (hsa-mir-106a, hsa-mir-145, hsa-mir-126, hsa-mir-17) associated with it. The remaining 491 candidate miRNAs are sent to SMALF for prediction. The results are shown in Table 6. Our study results show that all the top ten miRNA candidates about colon cancer are verified in MNDR v3.0 or miRCancer.

The third disease we studied was breast cancer. The number of people who have breast cancer is increasing since the 1970s, and now it has become common cancer affecting women’s physical and mental health [40]. We remove 202 miRNAs (has-mir-1245a, has-mir-1245b, has-mir-1258, et al.) associated with breast cancer. There are 293 candidate miRNAs for breast cancer. The results are shown in Table 7. Our study results show nine of the top ten miRNA candidates about breast cancer are confirmed in MNDR v3.0 or miRCancer. It’s worth noting that biological experiments haven’t validated hsa-mir-487b. It is likely associated with breast cancer.

## Conclusion

Discovering unknown miRNA-disease associations is vital for us to understand the pathogenesis of diseases at the molecular level. However, the biological experiment-based approach to uncovering unknown miRNA-disease associations is still very limited. Thus, it is increasingly important to use computational methods to predict unknown miRNA-disease associations. We developed SMALF, which is a computational method by combining similarity data and latent features. SMALF first extracted miRNAs and diseases latent features from the original miRNA-disease association matrix by utilizing a stacked autoencoder, respectively. Then, integrating miRNA functional similarities, disease semantic similarities, miRNA latent features, and disease latent features generated the feature vector representing miRNA-disease. Finally, SMALF obtains the prediction result by employing the XGBoost algorithm. We performed five-fold cross-validation experiments. SMALF achieved an AUC value of 0.9503, which is much higher than many other computational methods. Besides, the case studies also indicated that SMALF could infer unknown miRNA-disease interactions effectively. However, our work still has some room for improvement. Due to the lack of negative samples, we select unknown miRNA-disease associations as negative samples. There may be false negatives in these negative samples, which may also impact the experimental results. Therefore, finding reliable negative samples will help further improve the performance of the model.

## Methods

### Problem description

Researchers use lots of biological experiments to confirm miRNAs-disease associations, and by tapping the potential connections between human diseases and biomolecules, which could effectively boost the prevention, diagnosis, and treatment of human diseases. How to efficiently and accurately dig out the potential relationship between miRNA and disease is what we want to breakthrough. Most of the existing studies are based on the miRNA-disease databases provided by HMDD V2.0 [41]. To extract latent features of existing miRNA-disease associations, the known associations are identified by constructing an adjacency matrix Y. The research task of this paper is to discover the unobserved potential connections in known miRNA-disease association matrix(0 in matrix Y).

### Human miRNA-disease association

To express the relationship between miRNA and disease, the adjacency matrix Y of the interaction between miRNA and disease is constructed. If miRNA m(i) and disease d(j) have a known association in this matrix, the value of Y(i,j) at the corresponding position of the matrix is set to 1, otherwise to 0. Note that, in this association, the 0 matrix does not indicate that there is no relation between miRNA and diseases. It only indicates that potential links are not yet discovered. For the ideal experimental result, it is necessary to select the positive and negative samples of miRNA-disease association. During the experiment, we used the miRNA-disease associations that are the same as Zhou et al [22]. and its 5430 positive samples and 5418 negative samples.The statistical information of the dataset is shown in Table 8.

**Table 8:** Statistics of the constructed dataset

No. of miRNAs	No. of diseases	No. of known associations	Association density
495	383	5430	0.0286

### MiRNA functional similarity

According to previous research results, it is not difficult to find that miRNA functional similarity is often more likely to be associated with phenotypically similar diseases. The miRNA functional similarity score can be computed [42]. We can construct an adjacency matrix FS(m(i),m (j)) to point out the useful similarity between miRNAs with records.

### Disease semantic similarity

Inspired by previous studies, the MeSH database (http://www.ncbi.nlm.nih.gov/), which is widely used to obtain disease-related data, is extracted to constructa directed acyclic graphs(DAG). For the given D, DAG(D) = (D, T, E),where T(D) represents the node set composed of D and all of its ancestor nodes, and the parent node. The edge directly connected by the child nodes is defined as E(D). Finally, as Xuan et al [43], the value of d(a disease) to D (semantic value) can be defined as:

3 $$\begin{aligned} \left\{\begin{array}{{l}} {D{1_D}\left( d \right) = 1{{\,}}if{{\,}}d = D}\\ {D{1_D}\left( d \right) = max\left\{ \triangle {{{*}}D{1_{{\rm D}}}\left( {{{{\text{d}}^{\prime}}}} \right) |{{ {{{{\text{d}}^{\prime}}}}}} \in {{{\text{child}}\;{\text{of}}\;{\text{d}}}}} \right\} {{\,\,}}{\text{if}}{{\,}}{\text{d}}{{\,}} \ne {{\,}}D} \end{array}\right. \end{aligned}$$

where $\triangle$ is the semantic contribution attenuation factor. Xuan et al. denoted the value of $\triangle$ to 0.5, the contribution value of disease D to itself is 1, and the value of other diseases to D decreases as the distance. From the above formula of the semantic value:

4 $$\begin{aligned} DV\left( D \right) = \mathop \sum \limits _{d \in T\left( D \right) } {D_D}\left( d \right) \end{aligned}$$

if two diseases can share more DAGs, they will be able to obtain a higher semantic similarity value. Therefore, the semantic similarity score SS between two diseases is:

5 $$\begin{aligned} SS\left( {d\left( i \right) ,d\left( j \right) } \right) = \frac{{\mathop \sum \nolimits _{t \in T\left( {d\left( i \right) } \right) \cap T\left( {d\left( j \right) } \right) } \left( {{D_{d\left( i \right) }}\left( t \right) + {D_{d\left( j \right) }}\left( t \right) } \right) }}{{DV\left( {d\left( i \right) } \right) + DV\left( {d\left( j \right) } \right) }} \end{aligned}$$

### Stacked autoencoders for latent features of miRNAs and diseases

In the adjacency matrix Y constructed by human miRNA-disease associations, the known 5430 miRNA-disease associations account for only 2.8% of all disease-miRNAs. In order to better represent these sparse primitive simple data, The stacked autoencoder extracts the potential relationships contained in the high-dimensional and sparse original feature vectors of miRNA and disease.

Autoencoder(AE) is an unsupervised learning method. Its purpose is based on the input unlabeled data, through training to obtain a dimensionality reduction feature expression of the data after compression. The autoencoder is an artificial neural network composed of two sub-networks: encoder and decoder [44]. In this article, a stack encoder is used to extract potential associations of miRNA-disease. The stacked autoencoder is a cascade of multiple autoencoders, that is, contains multiple hidden layers to complete the task of extracting information layer by layer for the original features. The stacked autoencoder trains multiple layers of AE sequentially. After the first AE training is completed, the output of its encoder is used as the input of the second AE, and so on, and finally, a more representative and low-dimensional latent feature is obtained.

**Fig. 5:**
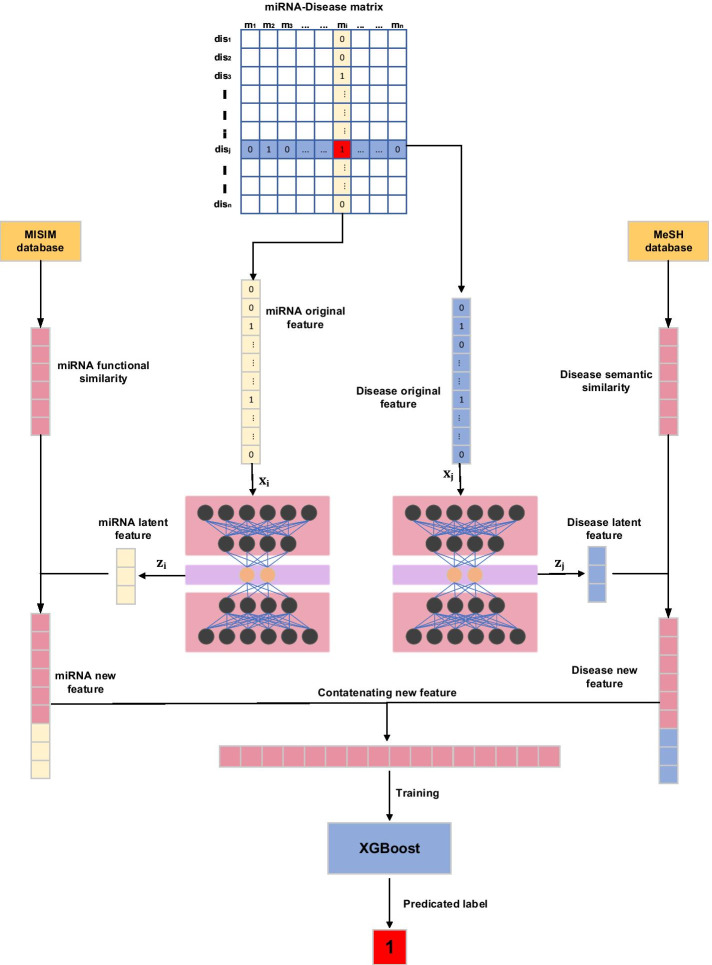
Overview of our proposed SMALF method for predicting miRNA-disease assoications.SMALF consists of four parts: Step1, we decompose miRNA-disease matrix *Y* into miRNA original feature *M* and disease original feature $D^T$. Step2, we utilize stacked autoencoders to learn the latent features of miRNAs and diseases from the original feature *M* and $D^T$. Step3,Integrating miRNA functional similarity, miRNA latent feature, disease semantic similarity, and disease latent feature generates the feature vector representing miRNA-disease. Step4, the XGBoost algorithm is employed to predict the miRNA-disease associations

### SMALF model

In this section, we will detail the SMALF model construction process, and show the overall process in Fig. 5.

#### Step 1: Matrix decomposition

Regarding the original matrix Y as the input, each row of Y is the original feature of the miRNA, and each column is the original feature of the disease. In the original feature vectors, m(i) and d(j) that decompose miRNA and disease, the one marked with 1 indicates that there is a correlation, and the one marked with 0 indicates that there is an unobserved correlation. Decompose miRNA disease association matrix Y into M and $D^T$.

6 $$\begin{aligned} Y\; = \;M{D^T} \end{aligned}$$

there $M,D^T \in Y^{m*n}$ is a real matrix. In our research, $M_i$ and $D_j^T$ are respectively regarded as the original feature vectors of m(i) and d(j).

#### Step 2: Extracts latent features by stacked autoencoders

In our autoencoder, the encoder H1 accepts the original feature m from miRNA in M and the encoder H2 accepts the original feature d from the disease in $D^T$ as input, define the i-th training sample $x_i=m$ in M in H1; define H2 The j-th training sample $x_j=m$ and encoder H extracts features from the low-dimensional code Z. The formula is as follows:

7 $$\begin{aligned}&{h_i}^{\left( l \right) }\; = \;{f_e}\left( {{W^l}{h_i}^{\left( {l - 1} \right) } + {b^l}} \right) \end{aligned}$$

8 $$\begin{aligned}&{z_i} = {W^L}{h_i}^{\left( {L - 1} \right) } + {b^L} \end{aligned}$$

where $l={1,....,L}$, we set L to 2, which means that use two hidden layers, $h_i^{(l)}$ is the l-th hidden layer, $h_i^{(0)}$ represents the input $x_i$ , $W^l$ is the weight matrix and $b^l$ is the bias of the l-th layer, The activation function $f_e (.)$ can effectively adjust the input through training.

The purpose of the decoder is to reconstruct the input $x_i$ as much as possible from the latent features $z_i$ output by the encoder. Its definition formula is as follows:

9 $$\begin{aligned}&{h_i}^{\left( l \right) }\mathrm{{\;}} = \mathrm{{\;}}{f_d}\left( {{W^l}{h_i}^{\left( {l - 1} \right) } + {b^l}} \right) \end{aligned}$$

10 $$\begin{aligned}&{\hat{x}_i} = {g_d}\left( {{W^L}{h_i}^{\left( {L - 1} \right) } + {b^L}} \right) \end{aligned}$$

Where $f_d (.)$ and $g_d (.)$ represent activation function and hyperbolic tangent function, respectively. where $f_d (.)$ and $g_d (.)$ represent activation function and hyperbolic tangent function, respectively.

Finally, the loss function is the sum of the reconstruction errors of all samples, and its expression is as follows:

11 $$\begin{aligned} L\;\left( {\begin{array}{*{20}{c}} {x,}&{\hat{x}} \end{array}} \right) = \;\mathop \sum \limits _{i = 1}^n {{||}}\left( {{x_i} - {\hat{x}}_i} \right) {{|}}{{{|}}^2}\; + \;\lambda \mathrm{{||}}{J_h}\left( {{x_i}} \right) \mathrm{{|}}{\mathrm{{|}}^2} \end{aligned}$$

among them, the first term loss is the square of the loss, the second term is the normalization of the Jacobian $J_h (x_i)$ and $\lambda$ is a hyperparameter. The stacked autoencoder will update the parameters of each node of the network iteratively to minimize the loss. it is trained through the iterative method of backpropagation, This step is also called fine-tuning. After continuous fine-tuning, the minimal loss is achieved, and the optimal solution of the autoencoder is reached. At this time, the latent feature z is the low-dimensional and high-density feature vectors $M_i$ and $D_j^T$ compressed by the miRNA and disease sparse features we need.

#### Step 3: Combining latent features and similarity features

So far, we have obtained the 64-dimensional miRNA and disease latent feature vectors $M_i$ and $D_j^T$ extracted by stacking autoencoder, which respectively concatenate with 495-dimensional miRNA functional similarity feature $FS_i$ and 383-dimensional disease semantic similarity feature $SS_j$ to new vectors that is 559-dimensional miRNA new feature and 447-dimensional disease new feature.

12 $$\begin{aligned} miRNA_{new}= & {} \left[ {\begin{array}{*{20}{c}} {{M_i}}\\ {FS_i} \end{array}} \right] \end{aligned}$$

13 $$\begin{aligned} Dis_{new}= & {} \left[ {\begin{array}{*{20}{c}} {{D_j}^T}\\ {SS_j} \end{array}} \right] \end{aligned}$$

then concatenate the two vectors to get a new vector for model prediction.

14 $$\begin{aligned} Vec_{new} = \left[ {\begin{array}{*{20}{c}} {miRNA_{new}}\\ {Dis_{new}} \end{array}} \right] \end{aligned}$$

#### Step 4: Predict new feature vectors by XGBoost

XGBoost accurately classifies the weak classifiers it contains through gradient iteration [45]. In this paper, we predict the new features of the miRNA-disease cascade in the new data set by the XGBoost model, which uses the cascaded $Vec_{new}$ as input and obtains its best gradient regression tree through training. XGBoost model contain K decision trees, $f_k$ represents the k-th decision tree, and the feature vector $Vec_{new\_i}$ is regarded as input $x_i$, and finally get the prediction result as the following formula:

15 $$\begin{aligned} \hat{y}_i^{\left( t \right) } = \mathop \sum \limits _k^K {f_k}\left( {{x_i}} \right) = \hat{y}_i^{\left( {t - 1} \right) } + {f_k}\left( {{x_i}} \right) \end{aligned}$$

where $\hat{y}_i^{(t)}$ means the classification result of the first j-th classifier, to minimize the loss of the objective function, the XGBoost algorithm adds a new function to the original model in each iteration. And use the function $\Omega (f_t )$ to control the complexity of the t-th subtree.

16 $$\begin{aligned} \Omega \left( {{f_t}} \right) = \gamma T + \frac{1}{2}\lambda \mathop \sum \limits _{j = 1}^T w_j^2 \end{aligned}$$

where T is the number of leaf nodes, $w_j$ is the score of each leaf node, $\gamma$ and $\lambda$ are the hyperparameters that control the proportion of complexity, and overfitting phenomenon can be prevented by adjusting these two hyperparameters. Furthermore, XGBoost also uses second-order Taylor expansion to optimize the objective function. The objective function of the t-th iteration is as follows:

17 $$\begin{aligned} \begin{array}{*{20}{l}} {obj^{(t) }} = \mathop \sum \limits _{i = 0}^m \left[ {{f_t}\left( {{x_i}} \right) {g_i} + \frac{1}{2}{{\left( {{f_t}\left( {{x_i}} \right) } \right) }^2}{h_i}} \right] + \Omega \left( {{f_t}} \right) \\ where\,{g_i} = \frac{{\partial l\left( {{y_i},\hat{y}_i^{\left( {t - 1} \right) }} \right) }}{{\partial \hat{y}_i^{\left( {t - 1} \right) }}},\;{h_i} = \frac{{{\partial ^2}l\left( {{y_i},\hat{y}_i^{\left( {t - 1} \right) }} \right) }}{{\partial {{\left({\hat{y}_i^{\left({t - 1} \right) }} \right) }^2}}} \end{array} \end{aligned}$$

where *l*(.) is the mean square error function of the iteration t-1, because $f_i(x_i)$ will finally be assigned to the leaf in the subtree, and its value can also be represented by the weight of the leaf $w_j$.

18 $$\begin{aligned} \begin{array}{*{20}{l}} {obj^{(t)}} = \mathop \sum \limits _{j = 1}^T [{w_j}\,{G_j} + \frac{1}{2}w_{j}^{2}({{H_j} + \lambda } )] + \gamma T \\ where{{\,}}{G_j} = \mathop \sum \limits _{i \in {I_j}} {g_j}, {H_j} = \mathop \sum \limits _{i \in {I_j}} {h_j} \end{array} \end{aligned}$$

where $I_j$ represents the sample set contained in leaf j. The iterative training of the above formula can effectively fit the new miRNA-disease features and obtain the optimal prediction model. Traverse all the data in the new test set, input the fused feature vector into the optimal SMALF model, and get the score prediction value for each potential miRNA-disease.

## Data Availability

The data and code used in the current study is available at:https://github.com/dayunliu/SMALF.

## Abbreviations

XGBoost: eXtreme Gradient Boosting
ROC: Rceiver operating characteristic
TPR: True positive rate
FPR: False positive rate
AUC: Area under ROC curve
Adaboost: Adaptive boosting
SVM: Support vector machine
RF: Random Forest
GBDT: Gradient Boosting Decison Tree
DAG: Directed acyclic graph

## References

[1] Ambros V (2001). micrornas: tiny regulators with great potential. Cell.

[2] Lee RC, Feinbaum RL, Ambros V (1993). The c. elegans heterochronic gene lin-4 encodes small rnas with antisense complementarity to lin-14. Cell.

[3] Ambros V (2004). The functions of animal micrornas. Nature.

[4] Bartel DP (2004). Micrornas: genomics, biogenesis, mechanism, and function. Cell.

[5] Erson A, Petty E (2008). Micrornas in development and disease. Clin Genet.

[6] Lynam-Lennon N, Maher SG, Reynolds JV (2009). The roles of microrna in cancer and apoptosis. Biol Rev.

[7] Calin GA, Dumitru CD, Shimizu M, Bichi R, Zupo S, Noch E, Aldler H, Rattan S, Keating M, Rai K (2002). Frequent deletions and down-regulation of micro-rna genes mir15 and mir16 at 13q14 in chronic lymphocytic leukemia. Proc Natl Acad Sci.

[8] Iorio MV, Ferracin M, Liu C-G, Veronese A, Spizzo R, Sabbioni S, Magri E, Pedriali M, Fabbri M, Campiglio M (2005). Microrna gene expression deregulation in human breast cancer. Can Res.

[9] Kozaki K-I, Imoto I, Mogi S, Omura K, Inazawa J (2008). Exploration of tumor-suppressive micrornas silenced by dna hypermethylation in oral cancer. Can Res.

[10] Masoudi MS, Mehrabian E, Mirzaei H (2018). Mir-21: a key player in glioblastoma pathogenesis. J Cell Biochem.

[11] Hébert SS, Horré K, Nicolaï L, Papadopoulou AS, Mandemakers W, Silahtaroglu AN, Kauppinen S, Delacourte A, De Strooper B (2008). Loss of microrna cluster mir-29a/b-1 in sporadic alzheimer’s disease correlates with increased bace1/β-secretase expression. Proc Natl Acad Sci.

[12] Chen X, Xie D, Zhao Q, You Z-H (2019). Micrornas and complex diseases: from experimental results to computational models. Brief Bioinform.

[13] Chen X, Liu M-X, Yan G-Y (2012). Rwrmda: predicting novel human microrna-disease associations. Mol BioSyst.

[14] Xuan P, Han K, Guo Y, Li J, Li X, Zhong Y, Zhang Z, Ding J (2015). Prediction of potential disease-associated micrornas based on random walk. Bioinformatics.

[15] Chen X, Yang J-R, Guan N-N, Li J-Q (2018). Grmda: graph regression for mirna-disease association prediction. Front Physiol.

[16] Jiang Y, Liu B, Yu L, Yan C, Bian H (2018). Predict mirna-disease association with collaborative filtering. Neuroinformatics.

[17] You Z-H, Huang Z-A, Zhu Z, Yan G-Y, Li Z-W, Wen Z, Chen X (2017). Pbmda: a novel and effective path-based computational model for mirna-disease association prediction. PLoS Comput Biol.

[18] Yao D, Zhan X, Kwoh C-K (2019). An improved random forest-based computational model for predicting novel mirna-disease associations. BMC Bioinform..

[19] Zheng K, You Z-H, Wang L, Zhou Y, Li L-P, Li Z-W (2019). Mlmda: a machine learning approach to predict and validate microrna-disease associations by integrating of heterogenous information sources. J Transl Med.

[20] Zhao Y, Chen X, Yin J (2019). Adaptive boosting-based computational model for predicting potential mirna-disease associations. Bioinformatics.

[21] Wang L, You Z-H, Chen X, Li Y-M, Dong Y-N, Li L-P, Zheng K (2019). Lmtrda: using logistic model tree to predict mirna-disease associations by fusing multi-source information of sequences and similarities. PLoS Comput Biol.

[22] Zhou S, Wang S, Wu Q, Azim R, Li W (2020). Predicting potential mirna-disease associations by combining gradient boosting decision tree with logistic regression. Comput Biol Chem.

[23] Zhang L, Chen X, Yin J (2019). Prediction of potential mirna-disease associations through a novel unsupervised deep learning framework with variational autoencoder. Cells.

[24] Xuan P, Sun H, Wang X, Zhang T, Pan S (2019). Inferring the disease-associated mirnas based on network representation learning and convolutional neural networks. Int J Mol Sci.

[25] Chen X, Huang L (2017). Lrsslmda: Laplacian regularized sparse subspace learning for mirna-disease association prediction. PLoS Comput Biol.

[26] Fu L, Peng Q (2017). A deep ensemble model to predict mirna-disease association. Sci Rep.

[27] Li J-Q, Rong Z-H, Chen X, Yan G-Y, You Z-H (2017). Mcmda: matrix completion for mirna-disease association prediction. Oncotarget.

[28] Zhao Q, Xie D, Liu H, Wang F, Yan G-Y, Chen X (2018). Sscmda: spy and super cluster strategy for mirna-disease association prediction. Oncotarget.

[29] Luo J, Xiao Q, Liang C, Ding P (2017). Predicting microrna-disease associations using kronecker regularized least squares based on heterogeneous omics data. Ieee Access.

[30] Gong Y, Niu Y, Zhang W, Li X (2019). A network embedding-based multiple information integration method for the mirna-disease association prediction. BMC Bioinform.

[31] Shin H-C, Orton MR, Collins DJ, Doran SJ, Leach MO (2012). Stacked autoencoders for unsupervised feature learning and multiple organ detection in a pilot study using 4d patient data. IEEE Trans Pattern Anal Mach Intell.

[32] Chen T, Guestrin C. Xgboost: a scalable tree boosting system. In: Proceedings of the 22nd ACM SIGKDD International Conference on Knowledge Discovery and Data Mining, 2016; 785–94.

[33] Xing, C., Chun-Chun, W., Jun, Y., Zhu-Hong, Y.: Novel human mirna-disease association inference based on random forest. Molecular Therapy Nucleic Acids 2018. 10.1016/j.omtn.2018.10.005 PMC6234518 30439645

[34] Ning L, Cui T, Zheng B, Wang N, Luo J, Yang B, Du M, Cheng J, Dou Y, Wang D. Mndr v3.0: mammal ncrna–disease repository with increased coverage and annotation. Nucleic Acids Research 2020. 10.1093/nar/gkaa707 PMC7779040 32833025

[35] Xie B, Ding Q, Han H, Wu D. Mircancer: a microrna-cancer association database constructed by text mining on literature. Bioinformatics. 2013. 10.1093/bioinformatics/btt014 23325619

[36] Ikura Y. Transitions of histopathologic criteria for diagnosis of nonalcoholic fatty liver disease during the last three decades. World J Hepatol. 2014. 10.4254/wjh.v6.i12.894 PMC4269908 25544876

[37] Xin WW, Hussain SP, Huo TI, Wu CG, Harris CC (2002). Molecular pathogenesis of human hepatocellular carcinoma. Toxicology.

[38] Parkin DM, Bray MF, Ferlay MJ, Pisani P (2005). Global cancer statistics, 2002. CA Cancer J Clin.

[39] Favoriti P, Carbone G, Greco M, Pirozzi F, Pirozzi REM, Corcione F (2016). Worldwide burden of colorectal cancer: a review. Updat Surg.

[40] Jemal A, Bray F, Center MM, Ferlay J, Forman D (2011). Global cancerstatistics. Ca Cancer J Clin.

[41] Yang L, Qiu C, Jian T, Geng B, Yang J, Jiang T, Cui Q. Hmdd v2.0: a database for experimentally supported human microrna and disease associations. Nucleic Acids Res. (D1), 1070, 2014. 10.1093/nar/gkt1023 PMC3964961 24194601

[42] Cui Q (2010). Inferring the human microrna functional similarity and functional network based on microrna-associated diseases. Bioinformatics.

[43] Xuan P, Han K, Guo M, Guo Y, Huang Y (2013). Prediction of micrornas associated with human diseases based on weighted k most similar neighbors. PLoS ONE.

[44] Ji C, Gao Z, Ma X, Wu Q, Zheng C. Aemda: inferring mirna-disease associations based on deep autoencoder. Bioinformatics. 2020. 10.1093/bioinformatics/btaa670 32726399

[45] Zhang Y, Chen J, Wang Y, Wang D, Cong W, Lai BS, Zhao Y, Sendiña-Nadal I. Multilayer network analysis of MIRNA and protein expression profiles in breast cancer patients. Plos One. 2019;14(4). 10.1371/journal.pone.0202311 PMC6448837 30946749

